# Nonmedical use of anxiolytics among Tunisian students: Connecting the dots from 2013 to 2021: Ahlem Silini

**DOI:** 10.1093/eurpub/ckac131.217

**Published:** 2022-10-25

**Authors:** A Silini, S Rejaibi, M Zid, N Zoghlami, I Ben Slema, R Mallekh, M Zribi, S Ben Youssef, N Ben Salah, H Aounallah-Skhiri

**Affiliations:** 1 Epidemiology Department, The National Health Institute, Tunis, Tunisia; 2 Faculty of Medicine, Tunis El Manar University, Tunis, Tunisia; 3 Nutrition Surveillance and Epidemiology, SURVEN, Research Laboratory, Tunis, Tunisia; 4 Intensive Care Unit Department, Center for Urgent Medical Assistance, Tunis, Tunisia

## Abstract

**Background:**

Nonmedical use of prescription drugs such as sedatives and anxiolytics is a fast growing public health threat in several countries. In Tunisia, although several studies have investigated the prevalence of nonmedical use of anxiolytics among school-age students, there is a gap in knowledge regarding trends in anxiolytics misuse. We aimed to determine the prevalence of nonmedical use of anxiolytics and examine trends between 2013 and 2021.

**Methods:**

Pooled data from three Mediterranean school surveys on alcohol and other drugs (MedSPAD I-2013, MedSPAD II-2017, MedSPAD III-2021) were used. Based on three-stage stratification sampling method, teenagers in first and second grades of secondary education, were enrolled. Were not included students enrolled in vocational training centers and out-of-school adolescents. Data collection was performed using a self-administered standardized questionnaire. We studied weighted lifetime nonmedical use of

prescription anxiolytics and performed global and by gender trend analysis. Epi data software was used for data entry and all statistical analysis, were performed with STATA software.

**Results:**

A total of 14.723 students were enrolled with sex ratio (Male/Female) equal to 0.61 and mean age of 16.2±0.8 years. The prevalence of nonmedical anxiolytics’ use increased from 2.1% to 3% then to 8.4% for 2013, 2017 and 2021, respectively. Global and by gender trends analysis concluded to significant increase from 2017 to 2021 (p < 10-3). However, a non-significant increase was revealed from 2013 to 2017.

**Conclusions:**

Our study is the first to confirm a significant increasing trend in non-medical use of anxiolytics among Tunisian adolescents. These findings emphasize the urgent need for early detection of psychological vulnerability among adolescents in order to prevent their engagement in such risky behaviors.

**Key messages:**

• Decision makers should be sensitized regarding the alarming increasing trend in non-medical use of anxiolytics, among Tunisian adolescents.

• The state control of these substances accessibility and early detection of psychological vulnerability, are highly required.

